# Exploring obesity phenotypes: a longitudinal perspective

**DOI:** 10.1007/s11154-025-09976-3

**Published:** 2025-06-18

**Authors:** Ricardo Rosero-Revelo, Mateo Tamayo, Ricardo Correa, Kevin M. Pantalone, David Creel, Bartolome Burguera, Marcio L. Griebeler

**Affiliations:** 1 Department of Endocrinology, Diabetes and Metabolism, Cleveland Clinic, Cleveland, OH USA; 2 Obesity Center, Fundación Santa Fe de Bogotá University Hospital, Bogotá D.C, Colombia

**Keywords:** Obesity phenotypes, Body composition, Bioelectrical impedance analysis, Metabolic health, Personalized intervention, BMI limitations

## Abstract

Traditional reliance on Body Mass Index (BMI) as a diagnostic tool for obesity is increasingly challenged due to its inability to differentiate between fat and lean mass and to capture fat distribution. Emerging evidence—including findings from our longitudinal study in Latino patients with obesity and insights from the 2025 Lancet Commission on Obesity—suggests that a comprehensive evaluation of body composition is essential for accurate risk stratification. This review synthesizes historical perspectives and recent developments in obesity phenotyping, detailing how the field has evolved from simple BMI-based assessments to multifaceted approaches incorporating bioelectrical impedance analysis (BIA) and supplementary anthropometric measures such as waist circumference and waist-to-hip ratio. We also examine the metabolic, genetic, and hormonal mechanisms underlying phenotypic variability, which help explain why individuals with similar BMIs may exhibit markedly different health risks. By integrating our original data with an extensive review of current literature, we demonstrate that refined obesity phenotyping can serve as an early indicator of progression from preclinical to clinical obesity. Such nuanced classifications offer the potential for more personalized therapeutic interventions aimed at optimizing weight loss outcomes and reducing cardiometabolic risk. Overall, our findings advocate for a multidimensional approach to obesity assessment that promises to improve clinical outcomes through tailored, phenotype-based strategies.

## Graphical Abstract

Panel (A) illustrates the evolution of obesity diagnosis, contrasting traditional anthropometric measurements with modern technologies like bioelectrical impedance analysis (BIA) for accurate body composition assessment. Panel (B) represents the conventional assumption that weight loss follows a predictable trajectory with proportional reductions in fat and muscle mass. Panel (C) showcases our longitudinal study findings in 709 Latino patients with obesity who underwent nutritional and exercise interventions, revealing four distinct phenotypic trajectories: Lean Preservers (49%), Mass Reducers (32%), Mass Retainers (10%), and Adipose Overload (9%), demonstrating the importance of dynamic body composition assessment beyond static BMI-based classification

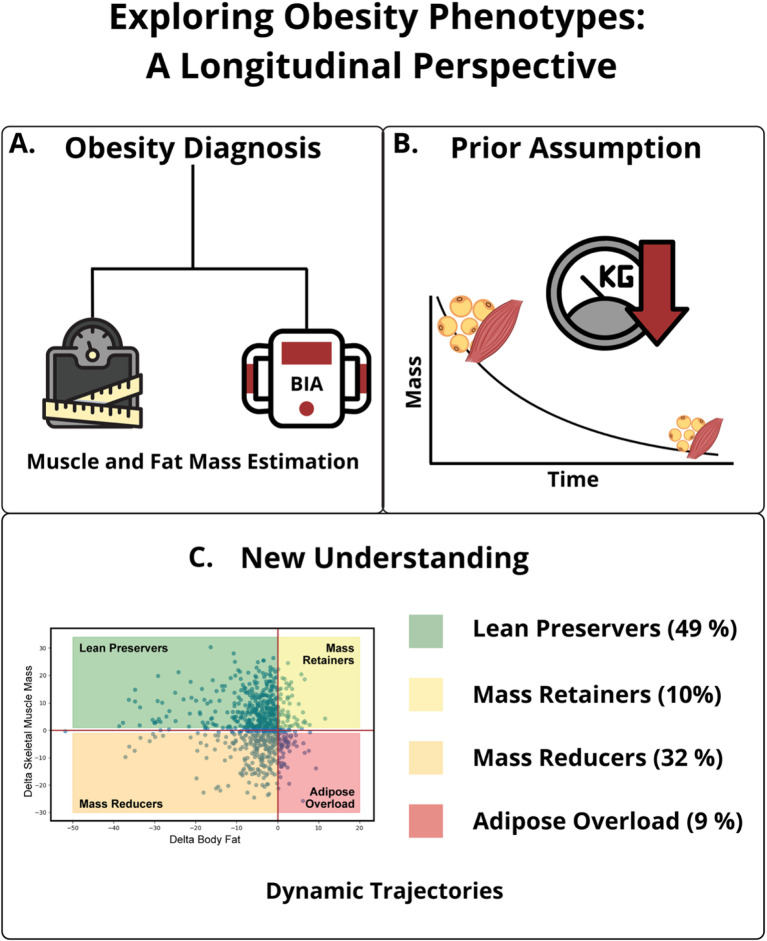

## Introduction

Obesity is a multifaceted chronic disease characterized by abnormal or excessive fat accumulation that poses significant health risks [1, 2]. For decades, Body Mass Index (BMI) has served as the primary metric for classifying obesity; however, BMI does not distinguish between fat mass and lean tissue, nor does it capture regional fat distribution—critical factors for assessing metabolic and cardiovascular risk [2–4]. Throughout the 20th century, various measures emerged to categorize populations in response to increasing mortality and healthcare costs linked to excess weight [5–7]. The 2025 Lancet Commission on Obesity, for instance, advocates for a nuanced diagnostic framework that differentiates between “preclinical obesity” (excess adiposity without evident organ dysfunction) and “clinical obesity” (excess adiposity associated with measurable impairments in tissue or organ function) relying on a body composition perspective confirmed by multiple measurement modalities [1].

Furthermore, criteria established by NHANES—which incorporate additional anthropometric measures such as waist circumference, waist-to-hip ratio, and waist-to-height ratio—underscore the limitations of relying solely on BMI [8]. Notably, the study identified sex-specific fat mass index (FMI) cut-off points (e.g., ≥ 8 kg/m² for men and ≥ 11 kg/m² for women), demonstrating significant differences by sex but no substantial variation by age or ethnicity [8]. Current research has demonstrated that obesity phenotypes, characterized by distinct patterns of body composition and metabolic profiles, can significantly influence treatment outcomes and long-term health risks. In this review, we synthesize the evolution of obesity phenotyping, integrate our original findings with current literature, and discuss the clinical implications of employing advanced body composition assessments for personalized treatment strategies.

## Evolution of obesity phenotyping

### Historical perspective

BMI, introduced by Quetelet in 1832, was based on the relationship between weight and the square of height [9]. Throughout the 20th century, various measures emerged to categorize populations in response to increasing mortality and healthcare costs linked to excess weight [5–7]. However, the term BMI, widely used today, was introduced by Ancel Keys in 1971, who also emphasized its limitations in assessing adiposity [10].

Is BMI still useful? The answer is more complex than a simple yes or no. While BMI remains a valuable and practical public health tool for large-scale decisions with acceptable accuracy [11], the rise of precision medicine and advanced, cost-effective tools has shifted the focus to body composition [1, 2, 12]. In this context, patients with the same BMI can exhibit vastly different health risks, depending on their fat distribution and muscle mass [13, 14]. The evidence behind phenomena such as the “obesity paradox” in cardiovascular outcomes further highlights the importance of assessing body composition [15, 16].

## Emergence of phenotypic classification

In response to these shortcomings, researchers have devised more sophisticated approaches to classify obesity by evaluating both fat and muscle compartments. Early contributions from Baumgartner and Hattori laid the groundwork for this approach, focusing on the differentiation of patients based on fat and muscle mass compartments, moving beyond BMI to offer a more detailed perspective of health risks [2, 17, 18]. This body composition–based framework expands our physiological understanding of obesity by delineating the differences between Metabolically Healthy Obesity (MHO) and Metabolically Unhealthy Obesity (MUO) [19–23]. In principle, these phenotypes correspond broadly with the latest definitions of “preclinical” and “clinical” obesity, respectively, emphasizing that excess adiposity, adipose tissue inflammation, and the ensuing metabolic derangements can occur independently of overall body weight [1, 2, 24–26].

**Fig. 1:**
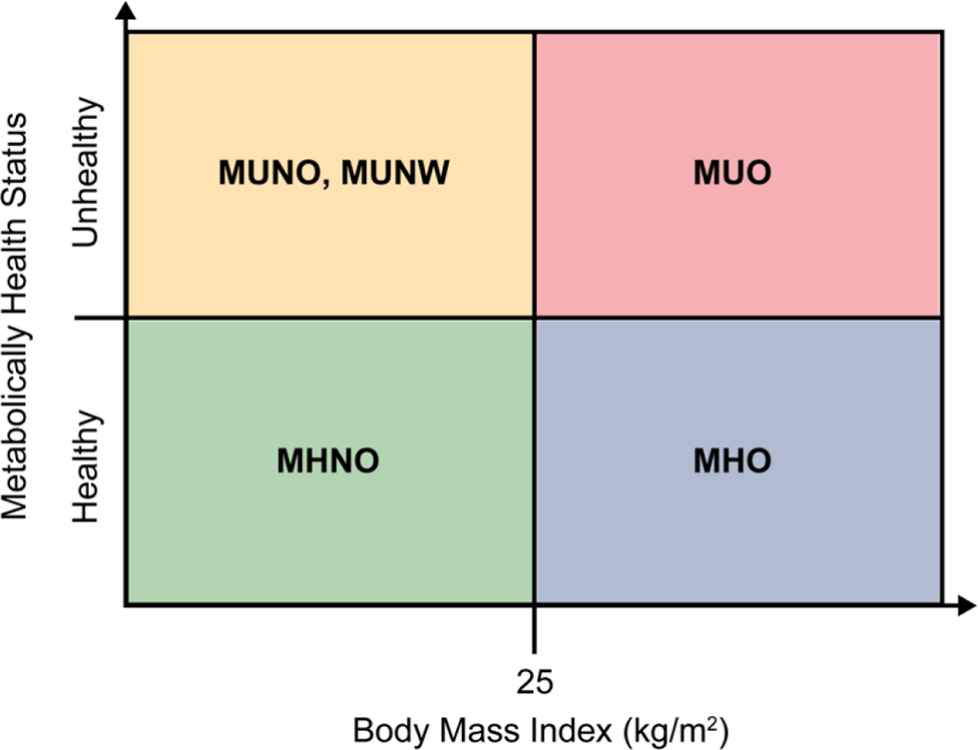
Classification of individuals based on body mass index (BMI) and metabolic health status. MUNO (metabolically unhealthy non-obesity), MUNW (metabolically unhealthy normal weight), MUO (metabolically unhealthy obesity), MHNO (metabolically healthy non-obesity), and MHO (metabolically healthy obesity) [14]

Recent evidence yields three pivotal insights. First, where fat is stored matters more than how much is stored: visceral, subcutaneous, and ectopic depots confer distinct metabolic risks [27–30]. Second, the fat-to-muscle balance is critical; loss of skeletal muscle magnifies the metabolic burden of adiposity and sharpens risk stratification [31–34]. Third, any obesity taxonomy must capture the specific metabolic and inflammatory derangements driven by elevated adiposity, not weight alone [1, 14]. Clinically, this perspective yields four metabolic phenotypes—Metabolically Healthy Obesity (MHO) and Metabolically Unhealthy Obesity (MUO) in both normal-weight and individuals with obesity [14]. Figure 1 overlays metabolic status onto BMI categories, demonstrating that patients with identical BMI values can display markedly different metabolic profiles and reinforcing the need for a multidimensional assessment framework in obesity care.

## Findings from our original research

Our longitudinal study involved 709 Latino patients who underwent a 12month intervention based solely on nutritional guidance and exercise, with no surgical or pharmacological treatments. The study identified four distinct phenotypic trajectories in terms of body composition changes.

These are defined as follows:

- ***Mass Reducers (Group A)***: Patients who lost both fat and muscle mass, serving as the reference group.
- ***Lean Preservers (Group B)***: Patients who achieved fat loss while gaining or maintaining muscle mass.
- ***Adipose Overload (Group C)***: Patients who gained fat while losing muscle mass.
- ***Mass Retainers (Group D)***: Patients who experienced increases in both fat and muscle mass.

These dynamic phenotypic categories are crucial for refined risk stratification. Table 1 presents the baseline characteristics and end follow-up data of the participants, delineating the group assignments, with each group letter corresponding to a specific phenotype.

**Table 1:** Baseline and Follow-Up characteristics

Baseline characteristics				At end of follow-up			
	Median, n	IQR, %		Median, n	IQR, %		P Value
Age	44 (y/o)	35–55					
Men	197	27.78%					
Women	512	72.21%					
Weight (Kg)	81.1	71.6–95		76.4	67.7–87.1		< 0.05
BMI (Kg/m^2^)	30.1	27.7–41.8		28.4	26–31.8		< 0.05
BFM (Kg)	33.8	27.7–41.8		29.2	23.9–36.5		< 0.05
FMI (Kg/m^2^)	12.8	10.2–16.2		11	8.9–14		< 0.05
FFM (Kg)	42	37.9–49.3		44.1	39.8–52.5		< 0.05
SSM (Kg)	22.9	20.4–27.3		24.1	21.5–29.2		< 0.05
PhA (^o^)	5.4	5–5.9		5.5	5–5.9		**0.45**
**Follow-Up**:							
Appointments				6	4–10		
Months				20	16–25		
A_Index				0.24	0.19–0.46		
Group A	-			227	32.02%		
Group B	-			344	48.52%		
Group C	-			64	9.03%		
Group D	-			74	10.44%		
BMI (A)	30.3 Kg/m^2^	27.5–34.9		28.2 Kg/m^2^	25.9–31.4		< 0.05
BMI (B)	30.3 Kg/m^2^	27.3–34.8		28.15 Kg/m^2^	25.1–31		< 0.05
BMI (C)	28.75 Kg/m^2^	26.8–31.8		29.85 Kg/m^2^	27.7–33		< 0.05
BMI (D)	28.75 Kg/m^2^	26.5–31.7		29.5 Kg/m^2^	26.9–33.1		< 0.05
FMI (A)	13.1 Kg/m^2^	10.5–16.5		10.9 Kg/m^2^	8.7–13.6		< 0.05
FMI (B)	13.2 Kg/m^2^	10.3–16.6		10.3 Kg/m^2^	8.5–13.4		< 0.05
FMI (C)	11.7 Kg/m^2^	10.4–14.6		12.9 Kg/m^2^	10.9–15.3		< 0.05
FMI (D)	11.20 Kg/m^2^	9.8–14		12.1 Kg/m^2^	10.4–15.7		< 0.05
SMM (A)	27.7 Kg	23.8–34.2		21.6 Kg	20–23.5		< 0.05
SMM (B)	21 Kg	18.8–23		26.9 Kg	23.6–33.4		< 0.05
SMM (C)	27.2 Kg	24.1–32.4		21.6 Kg	20.3–23.2		< 0.05
SMM (D)	21.3 Kg	19.6–23.9		26.5 Kg	23.7–31.3		< 0.05

Summary of demographic and body composition metrics. Abbreviations: BMI (Body Mass Index), BFM (Body Fat Mass), FMI (Fat Mass Index), FFM (Fat-Free Mass), SSM (Skeletal Muscle Mass), PhA (Phase Angle). Follow-up metrics include number of appointments, follow-up duration (months), adherence index (A Index), and group distributions with respective BMI changes

Table 1. Summary of demographic and body composition metrics. Abbreviations: BMI (Body Mass Index), BFM (Body Fat Mass), FMI (Fat Mass Index), FFM (Fat-Free Mass), SSM (Skeletal Muscle Mass), PhA (Phase Angle). Follow-up metrics include number of appointments, follow-up duration (months), adherence index (A Index), and group distributions with respective BMI changes.

## Body composition assessment

Accurate estimation of body composition is fundamental in both clinical and research settings, and a wide array of methodologies has been developed to address this need. In routine practice, techniques such as Dual-Energy X-Ray Absorptiometry (DXA) are among the most commonly employed due to their accessibility and robust assessment of bone and soft tissue compartments [8, 35, 36]. At the forefront of advanced methodologies, imaging modalities such as Computed Tomography (CT) and Magnetic Resonance Imaging (MRI) offer enhanced precision in quantifying adipose and lean tissues, while tracer techniques such as deuterium dilution provide an accurate measurement of total body water, which can be used to estimate lean mass [37–40]. Collectively, these diverse methods underscore the inherent heterogeneity in body composition among individuals and reflect a concerted effort to derive accurate estimates of fat and muscle distribution. In our study, we integrate these advanced approaches—particularly Bioelectrical Impedance Analysis (BIA)—to further refine obesity phenotyping and to provide a comprehensive understanding of metabolic risk.

## Bioelectrical impedance analysis (BIA)

Advances in technology have enabled more precise assessments of body composition. BIA permits the quantification of critical parameters such as fat mass, fat-free mass (FFM), and phase angle (PhA) [40–42]. This technique relies on measuring differences in electrical resistance and reactance, which reflect the varying conductive properties of fat, muscle, and other tissues [43]. By analyzing these impedance values, BIA provides an indirect yet reliable estimate of tissue composition and cellular integrity, a method that has been validated against other established tools such as DXA and MRI [40, 42]. In our study, we employed the SECA mBCA 514 device under standardized fasting conditions to ensure both reproducibility and reliability of the data [12, 44, 45].

### Comprehensive anthropometric and body composition assessment

**Fig. 2:**
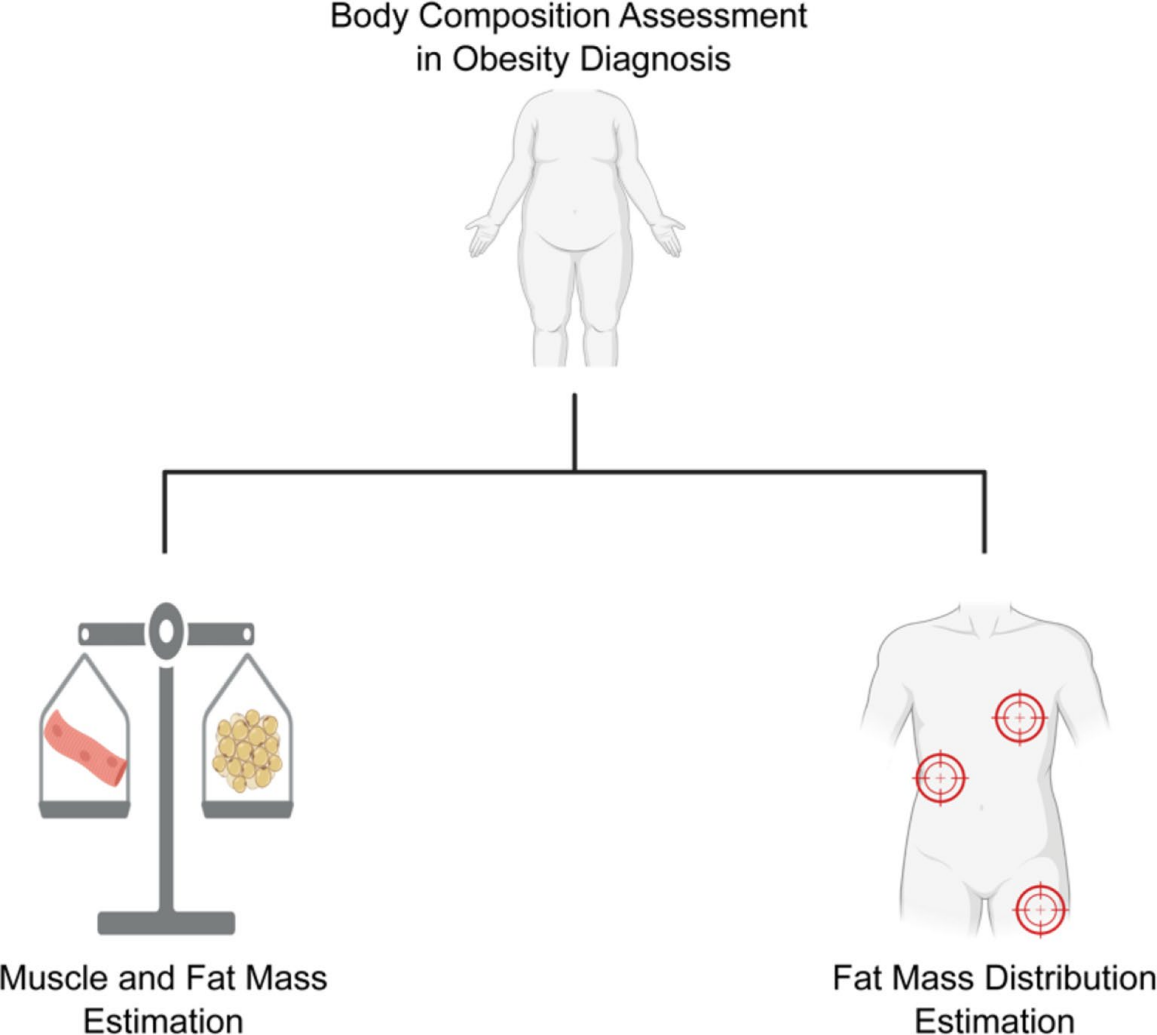
Body Composition Assessment in Obesity Diagnosis: A Dual Approach. Left (Bicompartmental Approach): Focuses on quantifying body composition by assessing the proportions of fat mass and muscle mass. This approach provides insight into the overall balance of tissue types within the body, helping to better understand the patient’s metabolic health. Right (Fat Distribution): Evaluates where the fat is distributed within the body, emphasizing the importance of differentiating between visceral, subcutaneous, and ectopic fat. This is critical for understanding the metabolic risks associated with obesity, as fat distribution plays a key role in determining health outcomes

Contemporary guidelines recommend supplementing BMI with additional anthropometric measures, such as waist circumference, waist-to-hip ratio, and waist-to-height ratio [1]. These measurements enhance the detection of visceral and ectopic fat—factors more closely linked to metabolic complications [27, 29, 30, 46]. The combined use of these complementary anthropometric measures with advanced techniques like BIA offers a comprehensive evaluation of body composition, which is essential for precise obesity phenotyping [45]. As depicted in Fig. 2, this dual approach integrates a bicompartmental assessment (quantifying fat and muscle mass) with an evaluation of fat distribution, thereby providing critical insights into metabolic risk and enabling more tailored therapeutic strategies.

## Obesity phenotyping

### Clinical implications of obesity phenotyping

In our study, we implemented a multinomial logistic regression analysis using the Mass Reducers group as the reference. Our analysis revealed that higher baseline fat-free mass (FFM) is linked to a lower likelihood of being classified as Lean Preservers (Group B), while robust adherence to treatment—as measured by our A-Index—significantly reduces the risk of transitioning to the Adipose Overload phenotype (Group C). These findings underscore the pivotal roles of both initial body composition and patient engagement in determining therapeutic outcomes. Detailed outcomes from these measurements are presented in Table 2.

**Table 2:** *Multinomial regression analysis*

Multinominal Regression				
	Coef	P-Value	OR	IC
**Group B**				
BFM	-0.0208	0.052	0.979	0.959–1.000
FFM	-0.169	0.000	0.845	0.812–0.878
PhA	-0.4305	0.054	0.650	0.419–1.001
Age	0.0208	0.041	1. 021	1.001–1.041
Sex	-0.0782	0.790	0.925	0.520–1.642
A_Index	0.8807	0.122	2.413	0.789–7.373
**Group C**				
BFM	-0.0381	0.022	0.963	0.931–0.995
FFM	-0.0314	0.107	0.969	0.932–1.007
PhA	0.2403	0.417	1.272	0.712–2.271
Age	-0.007	0.600	0.993	0.968–1.019
Sex	-1.0046	0.021	0.367	0.155–0.862
A_Index	-4.4483	0.000	0.012	0.001–0.099
**Group D**				
BFM	-0.0659	0.000	0.936	0.904–0.969
FFM	-0.1492	0.000	0.861	0.820–0.904
PhA	-0.7278	0.028	0.483	0.253–0.922
Age	0.0099	0.481	1.009	0.983–1.038
Sex	-0.3575	0.366	0.699	0.321–1.519
A_Index	-4.0162	0.000	0.018	0.002–0.153

Multinomial regression coefficients (Coef), p-values, odds ratios (OR), and confidence intervals (CI) for predictors of phenotype classification in Groups B, C, and D. Abbreviations: BFM (Body Fat Mass), FFM (Fat-Free Mass), PhA (Phase Angle), A Index (Adherence Index). Significant predictors include baseline body composition, age, sex, and adherence index, varying across groups

Table 2. Multinomial regression coefficients (Coef), p-values, odds ratios (OR), and confidence intervals (CI) for predictors of phenotype classification in Groups B, C, and D. Abbreviations: BFM (Body Fat Mass), FFM (Fat-Free Mass), PhA (Phase Angle), A Index (Adherence Index). Significant predictors include baseline body composition, age, sex, and adherence index, varying across groups.

Tailored interventions based on phenotypic classification have been demonstrated to enhance weight loss, improve cardiometabolic profiles, and reduce the incidence of obesity-related comorbidities [47–52]. In addition, factors such as genetic predispositions (e.g., MC4R gene variants), hormonal regulation (including insulin, leptin, and peptide YY), and the composition of the gut microbiome contribute to the variability in treatment responses [48, 53–55]. Such personalized therapeutic strategies promise more effective management of obesity compared to conventional approaches that rely solely on BMI.

**Fig. 3:**
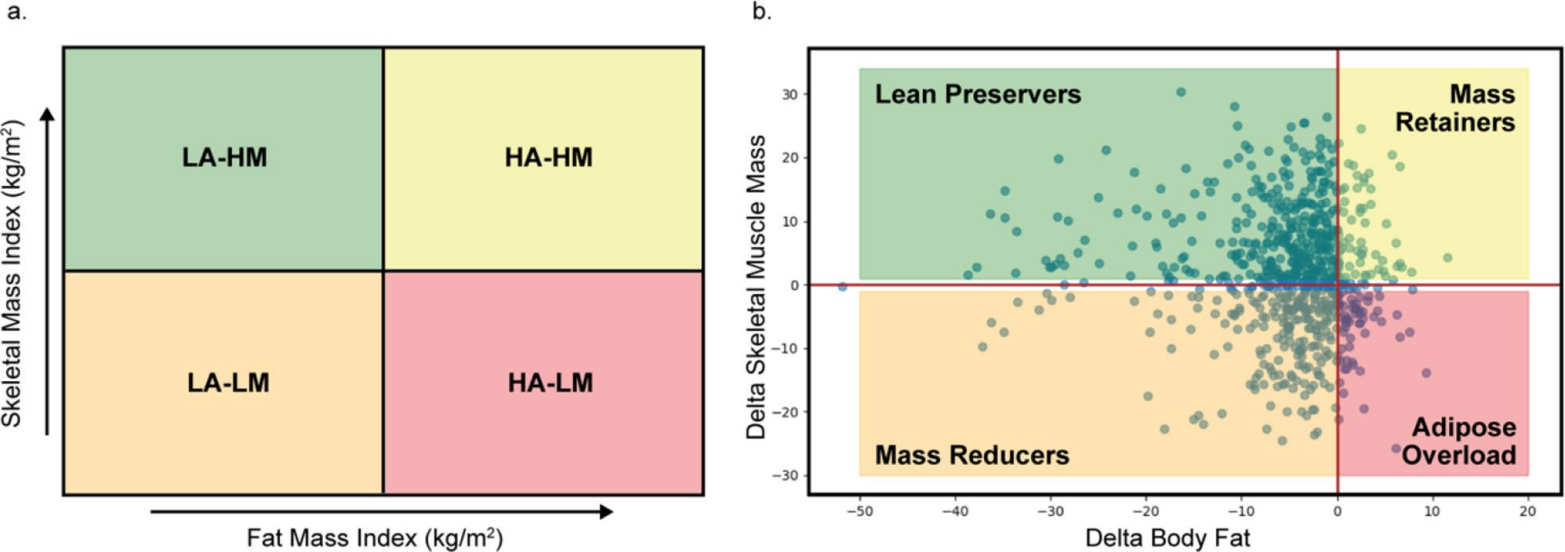
Body Composition Phenotypes. **(a)** Body-composition phenotype classification criteria by groups of **SMI** (Skeletal Muscle Index) and **FMI** (Fat Mass Index) [17, 18]. The quadrants represent four possible phenotypes: Low Adiposity - High Muscle Mass (LA-HM), High Adiposity - High Muscle Mass (HA-HM), Low Adiposity - Low Muscle Mass (LA-LM), and High Adiposity - Low Muscle Mass (HA-LM). **(b)** Proposed dynamic phenotypic trajectories during weight loss observed longitudinally: Lean Preservers (lose fat, gain muscle), Mass Retainers (gain both fat and muscle), Mass Reducers (lose both fat and muscle), and Adipose Overload (gain fat, lose muscle)

Our proposed phenotypes—Lean Preservers, Mass Retainers, Mass Reducers, and Adipose Overload—offer detailed insights into dynamic changes in body composition. These insights complement the new classification framework on Obesity, which distinguishes between “preclinical obesity” (excess adiposity without evident organ dysfunction) and “clinical obesity” [1]. Our findings suggest that the dynamic trajectories observed in our study could serve as early indicators of progression from preclinical to clinical obesity. For instance, patients classified as “Adipose Overload,” who experience fat gain coupled with muscle loss, may be at a higher risk of developing metabolic dysfunction and organ impairment, thereby aligning more closely with the concept of clinical obesity [1, 27, 56]. Conversely, the “Lean Preservers” phenotype, characterized by effective fat loss with muscle preservation or gain, may indicate a maintained or even improved metabolic state despite an elevated BMI—corresponding to a preclinical condition [2, 31, 32, 57]. Integrating these nuanced distinctions into the existing framework could improve the sensitivity and specificity of obesity classification, facilitating earlier and more targeted interventions. Figure 3 visually depicts these dynamic trajectories. This visual summary reinforces the heterogeneity of obesity and suggests potential pathways for personalized treatment strategies by linking phenotypic changes with clinical outcomes.

### Mechanisms underlying phenotypic variability

Understanding why obesity manifests so differently among individuals is a multifaceted challenge. A complex interplay of genetic, environmental, and behavioral factors contributes to the marked heterogeneity observed in obesity phenotypes [1, 46, 53, 54]. Genetic predisposition can influence metabolic pathways, adipocyte function, and even fat distribution patterns, thereby setting the stage for individual variability [24, 54, 58]. Environmental exposures—including diet, physical activity, and socioeconomic factors—further interact with these genetic factors to shape how and where fat is stored, whether as visceral, subcutaneous, or ectopic deposits [53, 59]. Additionally, differences in inflammatory responses, hormonal regulation, and insulin sensitivity add layers of complexity, leading to distinct metabolic profiles that may explain why some individuals develop metabolic complications while others do not [22, 23, 26, 60, 61]. Below is a breakdown of these mechanisms in a clear, step-by-step manner.

### Hormonal regulation

Recent endocrinology research has clarified the hormonal network that controls fat accumulation and overall energy balance [58, 62–64]. Insulin promotes glucose uptake and lipogenesis, but when tissues become resistant it instead channels surplus energy into adipose stores [65, 66]. Ghrelin heightens appetite, whereas adiponectin—released by fat cells—improves insulin sensitivity and reduces inflammation; its levels drop as body fat rises [67, 68]. The incretins GLP-1 and GIP boost glucose-dependent insulin secretion and enhance satiety, and newly developed dual incretin agonists show the advantages of targeting several metabolic pathways simultaneously [67]. Together, these hormones and related peptides coordinate energy intake, storage, and expenditure, shaping each individual’s risk of excess adiposity and its metabolic complications.

#### Metabolic adaptation

Refers to the regulation of the energy balance equilibrium through a complex interplay of hormonal, neural, and metabolic mechanisms [58, 69, 70]. Recent evidence indicates that during caloric restriction, the body initiates intricate regulatory responses that not only lower basal metabolic rate (BMR) but also adjust energy consumption and expenditure [71–73]. These adjustments are influenced by central nervous system inputs that modulate appetite and energy output, as well as by hormonal signals—such as insulin, leptin, ghrelin, GLP-1, and GIP—that work within a dual set-point framework to fine-tune energy homeostasis [74–77]. This multifaceted response, which can vary considerably among individuals, partly explains why some people experience significant reductions in BMR and muscle loss while others preserve or even gain muscle mass. Such variability in metabolic adaptation underscores the insidious nature of obesity, revealing it as a disorder characterized by persistent and complex disturbances in the body’s regulatory mechanisms, thereby presenting considerable challenges for long-term therapeutic intervention [78].

#### Genetic and epigenetic influences

Our genetic makeup plays a pivotal role in determining susceptibility to obesity. Recent research has reinforced that specific genetic variant—most notably within the FTO gene, as well as others such as MC4R—significantly affect fat storage, energy balance, and metabolic efficiency [53, 54]. In tandem, epigenetic modifications, including DNA methylation, histone modifications, and non-coding RNA activity, mediate the impact of environmental factors on gene expression. These dynamic epigenetic processes not only influence the risk of obesity development but also contribute to the varied responses observed in weight loss interventions [53, 55]. Collectively, these findings highlight a complex interplay between inherited genetic factors and environmentally driven epigenetic changes, underscoring the need for personalized therapeutic strategies in obesity management.

#### Muscle-fat interactions

A direct relationship exists between muscle and fat, governed by a complex network of regulatory signals including adipokines and myokines [31, 32, 34, 79]. According to Forbes’ theory, the interplay between these compartments is not linear but follows a sigmoid curve [80]. Moreover, the endocrine functions of adipose and muscle tissues, through the release of specific adipokines and myokines, intricately modulate anabolic and catabolic processes [81–84]. These regulatory mechanisms contribute significantly to the unique phenotypic patterns observed in obesity, emphasizing the importance of considering the dynamic balance between muscle and fat in therapeutic strategies.

#### Environmental and behavioral factors

Lifestyle choices, including dietary habits and physical activity, are pivotal in shaping obesity phenotypes [47, 59, 64, 85]. Adherence to a balanced diet and regular exercise not only supports the maintenance of muscle mass but also facilitates fat loss. Detailed dietary records and validated physical activity assessments offer valuable insights into these behaviors, which significantly influence an individual’s metabolic profile [52]. By integrating these environmental and behavioral dimensions with biological mechanisms, clinicians can gain a more comprehensive understanding of the multifactorial nature of obesity and develop tailored treatment strategies that address the unique needs of each patient.

### Limitations and future directions

Despite the promising insights provided by our multidimensional approach to obesity phenotyping, several limitations must be acknowledged. Our study was conducted exclusively within a Latino cohort, which may limit the generalizability of our findings to other populations. Additionally, the retrospective design and the reliance on follow-up attendance—as measured by our A-Index—may not fully capture the complete spectrum of patient adherence and behavior during the intervention. Another significant limitation is the absence of comprehensive metabolic data. Without detailed markers such as blood glucose, lipid profiles, and blood pressure measurements, it is challenging to fully correlate the identified phenotypes with metabolic risk. This gap underscores the need for future studies to incorporate a broader range of biochemical and clinical parameters.

Looking forward, there is a clear need for prospective, multicenter studies that integrate detailed metabolic, biochemical, and genetic data to validate and refine obesity phenotyping systems. Enhanced measures of adherence—such as 24–72 h dietary recalls and validated physical activity questionnaires—should be incorporated to provide a more comprehensive understanding of patient behavior. Furthermore, future research should adopt a multidisciplinary approach, integrating genetics, epigenetics, and behavioral sciences, to further elucidate the determinants of obesity phenotypes and to develop more precise, personalized intervention strategies.

### Clinical implications

Embedding longitudinal body-composition phenotyping into routine obesity care shifts clinical focus from static BMI checkpoints to trajectory-based decision-making. By classifying patients as Lean Preservers, Mass Reducers, Mass Retainers, or Adipose Overload, clinicians can quickly discern whether weight change reflects true fat loss, adverse muscle loss, or hidden fat gain. Early detection of the Adipose Overload trajectory should trigger an immediate escalation or repositioning of nutritional, exercise, and pharmacologic interventions to avert cardiometabolic decline, whereas identifying Lean Preservers justifies intensifying obesity therapy while simultaneously safeguarding—or even augmenting—skeletal muscle. Incorporating these phenotypes into clinical guidelines could sharpen risk stratification, optimize resource allocation, and support more precise, patient-centred care, while also laying the groundwork for studies on the biological origins of divergent trajectories and for trials of phenotype-guided therapies.

## Conclusions

Relying solely on BMI obscures the biological and clinical heterogeneity of obesity. A multidimensional assessment—combining advanced body-composition analysis with complementary anthropometric and metabolic markers—offers a far more accurate framework for risk stratification and personalized care. Our longitudinal findings, together with the broader evidence base, endorse this paradigm shift and show that early, phenotype-driven insights can flag progression from pre-clinical to clinical obesity. Embedding trajectory-based phenotypes (Lean Preservers, Mass Reducers, Mass Retainers, Adipose Overload) into future guidelines will translate these insights into practice, enabling clinicians to intervene on meaningful body-composition changes rather than weight alone and ultimately improve cardiometabolic outcomes. Future research should unravel the biological, behavioural, and environmental determinants that govern selective gains and losses in fat and muscle compartments, paving the way for truly phenotype-guided prevention and treatment strategies.

## Data Availability

No datasets were generated or analysed during the current study.
